# Minimal Important Difference of the Psychosocial Impact of Dental Aesthetics Questionnaire Following Orthodontic Treatment: A Cohort Study

**DOI:** 10.3390/children9040506

**Published:** 2022-04-03

**Authors:** Wan Nurazreena Wan Hassan, Mohd Zambri Mohamed Makhbul, Zamros Yuzadi Mohd Yusof, Siti Adibah Othman

**Affiliations:** 1 Department of Paediatric Dentistry and Orthodontics, Faculty of Dentistry, Universiti Malaya, Kuala Lumpur 50603, Malaysia; sitiadibah@um.edu.my; 2 Orthodontic Unit, Klinik Pergigian Cahaya Suria, Pudu Sentral, Ministry of Health Malaysia, Kuala Lumpur 55100, Malaysia; dr.zambri@moh.gov.my; 3 Department of Community Oral Health & Clinical Prevention, Faculty of Dentistry, Universiti Malaya, Kuala Lumpur 50603, Malaysia; zamros@um.edu.my

**Keywords:** orthodontic appliances, fixed, index of orthodontic treatment need, minimal clinical important difference, validation study

## Abstract

(1) This study aimed to do a longitudinal validation of the psychosocial impact of dental aesthetics questionnaire (PIDAQ) and determine its minimal important difference (MID) following orthodontic treatment; (2) 42 adolescents (11 to 18 years old) were recruited for treatment with fixed appliances and the participants answered the Malaysian PIDAQ prior to treatment (T0), mid-treatment (T1), and post-treatment (T2), plus a global health transition scale at T1 and T2. Data analyses included repeated measures ANOVA and paired sample *t*-tests. Test–retest was administered 2 to 6 weeks from T0; (3) The final sample comprised 37 subjects (response rate = 88.1%). The 95% limits of agreement were −10.3 to 8.5 points. In the anchor-based approach, those who experienced the smallest changes by reporting their dental aesthetics as “a little improved” had an insignificant PIDAQ score change of −5.3 at T1. At T2, the PIDAQ scores of those in this category were reduced significantly (mean change = −26.2; effect size (ES) = 1.0; *p* = 0.34). In the distribution-based approach, standardized PIDAQ scores were significantly reduced, with medium (0.7) to large (1.5) ES at T1 and T2, respectively; (4) The PIDAQ can detect orthodontic-related changes in patients’ psychosocial well-being. The post-treatment MID is 26 scale points with large ES.

## 1. Introduction

Malocclusion is a condition of unaesthetic dental arrangement that psychosocially impacts young people with increasing severity [1]. It is common, with a 46% prevalence of orthodontic treatment needs among adolescents worldwide [2]. Improving quality of life through correction of the malocclusion is one of the aims of orthodontic treatment.

The impacts caused by the malocclusion can be measured using oral health related quality of life (OHRQoL) instruments. The instrument needs to be longitudinally validated to be able to detect orthodontic-related changes in a patient’s well-being during treatment. The minimal important difference (MID) is the smallest change in an outcome score that is considered important to patients [3], and this would provide a meaningful threshold for clinicians to determine if their patients had benefited from the treatment. 

The psychosocial impact of dental aesthetics questionnaire, PIDAQ, is a conditioned-specific OHRQoL instrument that was developed to measure psychological and social impacts related to malocclusion [4]. It has been cross-culturally adapted in many languages for use by adolescents [5,6,7,8,9,10] who commonly seek orthodontic treatment. Cross-sectional studies on adults showed that the PIDAQ scores after orthodontic treatment were lower than those with malocclusions but have yet to receive orthodontic treatment [11]. A prospective study also found that the PIDAQ scores of adolescents and young adults significantly reduced after one year of fixed appliances therapy [12]. However, the longitudinal validity of PIDAQ for use by Malaysian adolescents has yet to be reported.

The minimal important difference (MID) is defined as “the smallest difference in score in the domain of interest that is considered to be clinically meaningful, which patients perceive as beneficial and which would mandate, in the absence of troublesome side effects and excessive cost, a change in the patient’s management” [13]. MID can be determined primarily by an anchor-based approach and supported by a distribution-based approach. The anchor-based approach links the change of the OHRQoL instrument to an interpretable anchor of an independent variable with recognised properties to provide the meaning of a specific degree of change, while the distribution-based approach relates changes to treatment to certain measures of variability [13].

The MID of the PIDAQ has yet to be evaluated in any setting. Thus, the objectives of this study were to evaluate the sensitivity and responsiveness of the Malaysian PIDAQ to fixed orthodontic treatment and to determine its MID. The outcome of the study will provide MID values for the Malaysian PIDAQ that would facilitate the interpretation of changes in scores to evaluate treatment effectiveness.

## 2. Materials and Methods

### 2.1. Study Design

This was a cohort study involving adolescent patients that was undertaken at an orthodontic clinic of an academic institution and a government-based orthodontic clinic in Kuala Lumpur, Malaysia, from June 2016 to April 2021. Subjects were recruited by consecutive clinical convenient sampling from the waiting list. The inclusion criteria were adolescents between 10 to 19 years old, as defined by the World Health Organization [14]. Patients with a history of orthodontic treatment, craniofacial syndromic patients, and subjects with learning difficulties who were unable to read written instructions in Malay or English were excluded. 

A sample estimation was determined using G*Power 3 software, which determined that a sample size between 27 to 35 was required to observe a change of a moderate effect (η^2^ = 0.06) [15] with an α of 5% and power of 80% to 90%, respectively. This was increased to 42 subjects to mitigate against a potential 20% loss to follow-up.

Subjects and their parents were briefed on the nature of the study and written informed consent was obtained prior to recruitment. Subjects underwent orthodontic treatment as judged by their attending clinicians, which included extractions and other adjunct appliances to fixed orthodontic appliances. Subjects completed questionnaires prior to treatment (T0), mid-treatment (T1), and post-treatment (T2). T1 was determined after the levelling and alignment phase upon reaching the working archwire of 0.019″ × 0.025″ stainless steel. At this stage, the displacements and rotations had been corrected and the incisors had been gathered to close any anterior spacing. Following this stage, patients continued treatment for overbite and overjet corrections, closure of the remaining spaces, and detailing. Once treatment was completed and the treatment aims achieved as judged by their clinicians, the fixed orthodontic appliances were debonded. T2 was immediately after debonding and prior to issuance of the retainers.

Prior to treatment (T0), subjects answered the self-administered questionnaire in the waiting room, and this comprised their demographic information, a global rating of perceived dental appearance, and a validated PIDAQ prepared bilingually in Malay [16] and English [17]. The bilingual PIDAQ will subsequently be referred as the Malaysian PIDAQ. The questionnaires at T1 and T2 comprised the Malaysian PIDAQ, a global rating of perceived dental appearance, and a global health transition scale which assessed their perception of change in their dental aesthetics.

The Malaysian PIDAQ is a 22-item questionnaire measuring the dental self-confidence (DSC, 6 items), social impact (SI, 8 items), psychological impact (PI, 6 items), and aesthetic concern (AC, 2 items) of patients [16,17]. The response options were “not at all”, “a little”, “somewhat”, “strongly”, and “very strongly” and scored from 0 to 4, accordingly. The scores of each subscale were tabulated and the PIDAQ score is the tabulation of the scores of the three negative domains (SI, PI, and AC) and the reverse score of the positive DSC domain. Higher PIDAQ, SI, PI, and AC scores, and a lower DSC score indicate poorer impacts due to the dental appearance and vice versa. The global rating of perceived dental appearance comprised of a single item question, asked at T0, T1, and T2, for the patient to relate their general feeling about the appearance of their teeth at the current phase. The response options were “very poor”, “poor”, “average”, “good”, and “excellent.” The global health transition scale was used as the gold standard to evaluate the responsiveness to change of the Malaysian PIDAQ. It comprised of a single item question, asked at T1 and T2, for the patient to relate their feeling about the appearance of their teeth in relation to pre-treatment. The response options were “worse”, “a little worse”, “no change”, “a little improved”, and “much improved”.

The pre-treatment and post-treatment study models were used to assess the index of orthodontic treatment need (IOTN) [18]. The examiner (WNWH) was assessed for reliability in the use of the IOTN on 20 sets of study models, with another examiner (MZMM) for inter-operator reliability and after at least two weeks for intra-operator reliability. The intra-operator and inter-operator kappa values in the use of the dental health component of the IOTN (IOTN-DHC) were 0.82 (SD 0.11) and 0.83 (SD 0.12), respectively, indicating strong agreements [15]. The intraclass correlations for absolute agreement by two-way random effects model in the use of the aesthetic component of the IOTN (IOTN-AC) showed excellent agreements [19] at 0.95 (95% CI 0.88 to 0.98) for both intra-operator and inter-operator reliability.

### 2.2. Statistical Analyses

Data were analysed using IBM^®^ SPSS^®^ Statistics 26.0 (IBM Corp., New York, NY, USA). The Shapiro–Wilk test confirmed the majority of the PIDAQ scores, in total and by domain, were normally distributed (*p* > 0.05). Therefore, parametric analyses were employed. 

One patient dropped out early because she entered boarding school. One patient did not complete the questionnaire at T1 and was excluded from the study. Three patients were excluded from the study due to slow accrual. These patients consistently missed their review appointments and treatment times exceeded 36 months. The remaining subjects answered all items.

#### 2.2.1. Sensitivity

The sensitivity of the Malaysian PIDAQ was measured by assessing changes in the standardized scores from T0 to T2. The standardized scores were derived by the following formula: z = (x) [100/(m × k)]. Here, x = sum score, m = number of items, and k = response category [20]. The repeated measures ANOVA, followed by post hoc pairwise comparison, were used to assess the statistical significance of differences between the standardized scores of the Malaysian PIDAQ and its subscales over time. A negative score implies an improvement while a positive score implies a deterioration in the adolescent’s subjective oral impacts, except for DSC where change interpretation is reversed. The standardized effect size (ES) of the post hoc pairwise comparisons by Bonferroni correction was calculated by dividing the mean change score of the preceding stage of treatment subtracted from the proceeding stage of treatment by the standard deviation of the preceding stage of treatment mean [13]. The standardized ES was interpreted according to Cohen (1988) [15]. 

The sensitivity of the PIDAQ was also investigated by evaluating changes in the PIDAQ scores by normative need. Subjects were considered as having a normative need if the IOTN-DHC score was grade 5 (indicating very great need), grade 4 (indicating great need), or grade 3 (indicating borderline need) with an IOTN-AC score of 6 and above [21]. Subjects were considered as not having a normative need when the IOTN-DHC score was grade 1 (indicating no need), 2 (indicating slight need), or grade 3 with an IOTN-AC score of 5 and below. The repeated measures ANOVA, followed by post hoc pairwise comparison by Bonferroni correction, were used to assess the statistical significance of differences between the PIDAQ scores by normative need. Independent *t*-tests were used to assess differences in PIDAQ scores between the group with normative need and the group without normative need, measured at each time point.

The association of the changes in PIDAQ scores with the global rating of perceived dental appearance was assessed using the Friedman test. ES was calculated by the z-statistics divided by the square root of the number of observations and interpreted according to Cohen (1988) [15].

#### 2.2.2. Responsiveness

The responsiveness to changes in the PIDAQ score was assessed by comparing the change in scores based on the global health transition scale. In each category, the differences between the PIDAQ scores obtained at T0 and T1, and between PIDAQ scores obtained at T0 and T2 were assessed using paired *t*-test. The ES of the *t*-test was interpreted according to Cohen (1988) [15].

Subjects were invited to answer the PIDAQ again at least 2 weeks (T_R_) from T0 to assess to prevent recall bias when assessing for absolute measurement error, but no longer than 6 weeks to ensure that no clinical change had occurred [22]. Those who answered beyond this test–retest period were excluded. The absolute measurement error was analysed by the Bland and Altman 95% limits of agreement using MedCalc^®^ Statistical Software version 20.027 (MedCalc Software Ltd., Ostend, Belgium).

#### 2.2.3. Determining the Minimal Important Difference (MID)

The MID for the Malaysian PIDAQ was determined using both anchor-based and a distribution-based approaches. In the anchor-based approach, the anchor was derived from a patient-based non-clinical outcome; that is, using the global health transition scale. In this scale, patients who reported a small gain or loss in oral impacts are then identified as undergoing a change, which the subjects considered as minimally important to them. The MID was calculated by the mean change in outcome measures from T0 for subjects who reported to experience small changes from the treatment, taken as the responses of “a little worse”, “no change” and “a little improved” categories of the global health transition scale. In this study, only the category “a little improved” was used as the anchor as none of the subjects reported “a little worse” or “no change” at T1 and T2. The MID should be more than the absolute measurement error for a meaningful evaluative purpose [22]. 

In the distribution-based approach, the standardized effect size was used, which calculates the mean change as a ratio of the standard deviation. The relative size of the change effect was inferred using the ES threshold levels described by Cohen (1988) [15], where 0.2 to less than 0.5 was considered as “small”, 0.5 to less than 0.8 as “moderate”, and those above 0.8 as “large” [15].

## 3. Results

Forty-two subjects were recruited. After considering dropout and missing data, 37 subjects were included in the analysis, giving a response rate of 88.1%. Table 1 shows the demographic characteristics, their PIDAQ scores and global health transition scale statistics. The mean age was 14.4 years (SD 2.0). The mean treatment duration was 25.6 months (SD 9.1). 

Thirty subjects were included in the test–retest analysis for the absolute measurement error. The 95% limits of agreement of the PIDAQ scores were between −10.3 and 8.5. The limits of agreement for the subscales were −5.3 to 5.6 for DSC, −6.5 to 6.4 for SI, −5.2 to 4.4 for PI, and −1.8 to 1.3 for AC.

### 3.1. Sensitivity

Repeated measures ANOVA indicated a significant improvement in the standardized PIDAQ scores and its subscales (Table 2; *p* < 0.05). Post hoc analysis showed that the scores of standardized PIDAQ and its subscales, except for DSC, were significantly reduced at T1 and T2 (*p* < 0.05, 95% CI did not include zero), while for DSC, the scores were significantly increased at T1 (*p* < 0.001, 95% CI = 7.5–27.0) and T2 (*p* < 0.001, 95% CI = 10.1–34.0). The magnitude of change between T1 and T0 was small for PI and AC, medium for SI and PIDAQ, and large for DSC. The magnitude of changes between T2 and T0 were large.

Repeated measures ANOVA shows that both the normative need and non-normative need groups had significant differences in PIDAQ scores over time (Table 3). Post hoc pairwise comparisons showed that, for the non-normative need group, the reduction in PIDAQ scores were not statistically significant between all-time points. On the other hand, a significant reduction in PIDAQ scores between all-time points (i.e., between T1 and T0, between T2 and T0, and between T2 and T1) was observed in the normative need group.

The PIDAQ mean scores were not significantly different between the two groups at all time points, as determined by paired *t*-tests (Table 3). Nonetheless, the normative need group generally had higher PIDAQ scores at all time points compared to the non-normative need group. The differences were the highest at T0, followed by T1, and the smallest at T2.

The distribution of the PIDAQ scores ranked by the global rating of perceived dental appearance is shown in Table 4. At T0, the majority of subjects (94.6%) rated themselves as having very poor to average dental appearance, while only one subject each rated themselves as having good (2.7%) or excellent (2.7%) dental appearance. At T1, no subjects rated themselves as having very poor or poor dental appearance. The majority (64.9%) rated themselves as having good dental appearance, while the rest rated themselves as having average (16.2%) or excellent (18.9%) dental appearance. At T2, the subjects rated themselves as having good (54.1%) or excellent (45.9%) dental appearance only. The PIDAQ scores were also highest at T0, followed by T1, and were the lowest at T2. The Friedman test showed that the improvement in the perceived dental appearance was significantly different (*p* < 0.001). 

### 3.2. Responsiveness

Table 5 shows the responsiveness to change of the PIDAQ. At T1, less than half (*n* = 16; 43.2%) of the subjects reported that they perceived their dental aesthetics to be “a little improved”. At T2, subjects who reported their dental aesthetics as “a little improved” were fewer (*n* = 7; 18.9%). The majority of subjects reported their dental aesthetics to be “much improved” at T1 (*n* = 21; 56.8%) and at T2 (*n* = 31; 86.1%). None of the subjects reported no change or worsened dental aesthetics at T1 or at T2. At T1, subjects who reported their condition to be “a little improved” only had a significant increase in the DSC score with a medium ES. The change in scores of the PIDAQ and the other subscales were not statistically significant. At T2, subjects who reported their dental aesthetics to be “a little improved” had significant improvement in PIDAQ scores with a large ES and in the DSC scores with medium ES. The changes in the SI, PI, and AC scores were not significantly different. On the other hand, subjects who reported their dental aesthetics to be “much improved” had a significant reduction in the PIDAQ and its subscales’ scores, except for DSC, which had a significant increase. All the changes for the subjects in the “much improved” category showed a large ES at both T1 and T2.

### 3.3. Minimal Important Difference (MID)

In the anchor-based approach (taken from Table 5), those in the “a little improved” group were identified as undergoing a change after treatment which patients considered as minimally important to them. The change in scores for PIDAQ and its subscales at T1 were within the 95% limits of agreement. At post-treatment (T2), the MID for the PIDAQ and its subscales were satisfactorily larger than the 95% limits of agreement. The MID for the PIDAQ was rounded as 26 scale points, and for DSC the MID was rounded as 8 scale points (Table 6). However, the MID as measured by the change in scores between T0 and T2 for the other subscales (SI, PI, and AC) were not statistically significant. 

In the distribution-based approach (taken from Table 2), the MID for the PIDAQ and DSC at T2, based on the standardized ES was 1.5 and 2.2, respectively, which were considered as having large effect sizes. 

## 4. Discussion

This study examined the evaluative property of the Malaysian PIDAQ by measuring the responsiveness and interpretability of the instrument, as part of the quality criteria assessment for the measurement properties of health status questionnaires [22]. We demonstrated its potential use as a tool to detect changes in patients’ well-being in a clinical setting. Overall, our study showed that the Malaysian PIDAQ is sensitive and responsive to changes in adolescents’ psychosocial impacts following orthodontic treatment. The MID for PIDAQ was found to be 26 scale points with a large effect size.

Our findings are applicable to Malaysian adolescents in general, as the majority of the patients in our study were Malays, followed by Chinese and Indians, which reflects the demographics of the Malaysian population [23], and the proportion of younger and older aged adolescents was equal. The sample had a higher proportion of girls, who are common treatment seekers, as they are more conscious of their aesthetics than boys [24]. The results of the current study also implies the potential of other PIDAQ versions that followed similar adaptation process from the original construct, for use in assessing orthodontic-related changes of a patient’s well-being following fixed appliances therapy. 

Sensitivity analysis is a method to determine the robustness of an assessment by examining the extent to which results are affected by changes in methods, models, values of unmeasured variables or assumption [25]. In this study, the sensitivity of the PIDAQ to the expected changes following orthodontic treatment was tested by three methods: (1) assessing change in standardized PIDAQ scores, (2) assessing change in PIDAQ score by normative needs, and (3) assessing change in relation to the perceived dental appearance rating.

The pre-treatment mean PIDAQ score of 52.2 (SD 17.8) obtained in this study was higher compared to the mean of Malaysian schoolchildren, which was 32.6 (SD 16.2) [26]. This reflects different levels of impact due to malocclusion in adolescents within different settings. There was a significant improvement in PIDAQ scores during treatment, which further improved after treatment; a score of 25.6 (SD 0.9) in the present study was better than that of general Malaysian schoolchildren [26] and young adults [24], whose scores were 32.6 (SD 16.2) and 36.3 (SD 17.1), respectively (Table A1). This indicates improvement of the subjects’ well-being due to treatment to a level that was better than Malaysians of a similar age who had never received treatment. 

The current findings also show that the PIDAQ is sensitive to changes with respect to normative need and changes in perceived dental appearance following orthodontic treatment. The changes with respect to the normative need were apparent. Although subjects in both the normative need and in the non-normative need groups saw a significant reduction in PIDAQ scores, the reduction in scores as treatment progressed (T1) until completion (T2) was more apparent in those with normative need. The PIDAQ scores were higher before treatment in subjects with normative need than those without. However, as treatment progressed to completion, the differences between the two categories became less apparent as their malocclusion were corrected. In terms of changes in perceived dental appearance, most patients rated their dental appearance as very poor to average with high PIDAQ scores before treatment. As treatment continued, they rated themselves as having average to excellent dental appearance with lower PIDAQ scores. At the end of treatment, they only rated themselves as having either good or excellent dental appearance with even lower PIDAQ scores.

The responsiveness of an instrument is defined as its ability to detect clinically meaningful changes over time [22]. In this study, the proportion of subjects who reported their dental aesthetics to be “a little improved” was less than half of the total subjects (43.2%) mid-treatment, and the proportion further reduced to 18.9% post-treatment. Their PIDAQ scores did not change significantly during treatment. The change in PIDAQ and its subscales’ scores was generally not significant during treatment. After treatment, the change in scores were significant only for the PIDAQ and DSC scores, with large magnitude of change. On the other hand, subjects who reported “much improvement” during and after treatment showed significant improvement in the change in scores for the PIDAQ and its subscales. The changes were almost double or triple those whose dental aesthetics were “a little improved” during treatment but were almost similar after treatment. In terms of the magnitude of change, there was a clear gradient in the ES values across the categories of the global health transition scale from “a little improved” to “much improved” for the PIDAQ and its subscales. Thus, the study has shown that the change in mean score and effect sizes of the PIDAQ and its subscales changed as expected following the gradient of adolescents’ perceptions of their dental aesthetics after treatment. These findings provide evidence of the instrument’s responsiveness to change in dental aesthetics after treatment.

For the instrument to be able to have a meaningful responsiveness to change property, the absolute measurement error should be smaller than the MID. The MID is the smallest difference in score in the domain of interest that is considered to be clinically meaningful, which patients perceive as beneficial [13]. This study used the Bland and Altman method [27] for absolute measurement error, because 95% limits of agreement are easily interpretable [22]. The trend of the 95% limits of agreement of the PIDAQ subscales was similar to those reported in previous studies [16,17], whereby the SI had the widest range and AC had the narrowest range. This is likely because SI is comprised of the most items of eight, followed by DSC and PI equally with six items, and AC with two items. 

In this study, the change in scores during treatment for subjects who considered their dental aesthetics as “a little improved” was only significant for the DSC subscale. However, the change in DSC score was within the 95% limits of agreement, indicating it was not very different from the variability accounted by intra-measurement disagreement within a period of less than 6 weeks. Therefore, the differences may not be clinically meaningful, as the values were not distinguishable from measurement error. On the other hand, the change in scores after treatment for subjects who considered their dental aesthetics as “a little improved” was significant for the PIDAQ and DSC subscale with values that were beyond the range of their respective 95% limits of agreement. Thus, the differences in the score at the level of the MID were distinct from measurement error. Therefore, the MID of the Malaysian PIDAQ and the subscale DSC is measurable after fixed appliances therapy has been completed.

### 4.1. Limitations

Subjects comprised patients with varying malocclusions. Although they were all given fixed appliances, their treatment protocol was adjusted according to individual needs such as orthodontic adjuncts and extractions. The appliances and having extraction or non-extraction may have confounded the perception of dental aesthetics.

Change was measured up to post-treatment, which was immediately after debond. It is not known if the change experienced was transient or permanent. Long-term follow up is recommended to assess the effectiveness of treatment into the retention phase.

The effect size for the change in psychosocial impacts after fixed appliances has been estimated to be large [11], which would have only required a much smaller sample. However, a large sample size for determining the MID has been recommended to increase the chances of recruiting subjects who would report “small change” or “no change” for a better precision [13]. Consideration was made not to recruit a sample that was too large, as it may detect small differences that are trivial [28]. Thus, the sample size for this study was based on a medium effect size to balance these requirements. The precision of the MID may have been limited due to the small sample of subjects who reported their dental aesthetics as “a little improved”. Nonetheless, the effect sizes, particularly at post-treatment, were large even with a small sample, suggesting the power of the study was adequate. 

Comparing subjects by normative need added value in the sensitivity assessment of the instrument. However, patients with no normative need are not usually recommended for treatment. In this study, subjects in this category only comprised 21.6% of the sample. They comprised patients with bimaxillary proclinations and spacing, and such features of the malocclusion do not grade highly on the IOTN. Treatment was provided as such patients are affected by the negative perception of protrusive dentition/lips [29] and gaps between the teeth.

Recent systematic review found the most common method used to determine the MID of health-related quality of life instruments is the anchor-based approach using non-clinical anchors from patients’ perspectives [30]. The use of different ways to calculate the MID may lead to different estimations of the MID [30]. Therefore, clinicians should be mindful that the MID value should be applied within the context it was studied under. The MID for the Malaysian PIDAQ for other type of treatments can be investigated in future studies.

The anchor-based approach that uses the global health transition scale can be confounded by recall bias and response shift in the individual’s internal standards of measurement as the adolescents undergo maturity. Thus, the study followed the recommendation to support the outcome with a distribution-based approach to enhance interpretability [13].

Clinicians should be mindful that change following treatment is an individual experience. While the MID value can provide a useful guideline to assess treatment effectiveness, they should manage each patient according to their needs.

### 4.2. Recommendations

It is important to objectively assess the success of treatment outcome. Orthodontic indices such as the Peer Assessment Rating (PAR) considered a change of 22 PAR points to give a ‘great improvement’ [31] while the Index of Complexity, Outcome, and Need (ICON) considered an ICON score of less than 31 as a clinically acceptable result [32]. Findings from the current study suggest using the Malaysian PIDAQ, with an MID of 26-point change as the threshold, in supporting the success of clinical outcome from patients’ perspective. Future studies are recommended to assess the effectiveness of treatment outcome in reducing the psychosocial impacts due to dental aesthetics using PIDAQ at a larger scale, such as in government dental clinics.

## 5. Conclusions

Within the limitations, the Malaysian PIDAQ is sensitive to changes in psychosocial impacts experienced by adolescents over their dental arrangements. The instrument is also responsive to treatment with clinically meaningful changes after fixed appliance therapy. The MID value of the PIDAQ after fixed appliance therapy is 26-point change. The MID for the DSC subscale is 8-point change. Clinicians can consider this difference in score to reflect the perceived improvement in dental aesthetics and to support a successful treatment outcome.

## Figures and Tables

**Table 1:** Demographic characteristics of the Malaysian psychosocial impact of dental aesthetics questionnaire (PIDAQ) score and global health transition scale (*N* = 37).

		PIDAQ Scores			Global Health Transition Scale			
		T0	T1	T2	T1		T2	
Characteristic	*N* (%)	Mean (SD)	Mean (SD)	Mean (SD)	A Little Improved, *n* (%)	Much Improved, *n* (%)	A Little Improved, *n* (%)	Much Improved, *n* (%)
**Gender**								
Male	6 (16.2)	57.0 (13.1)	51.0 (23.8)	22.3 (10.9)	1 (6.3)	5 (23.8)	3 (42.9)	3 (10.0)
Female	31 (83.8)	51.3 (18.6)	37.9 (13.4)	26.3 (16.8)	15 (93.8)	16 (76.2)	4 (57.1)	27 (90.0)
**Age**								
11–14	19 (51.4)	49.3 (17.4)	37.2 (18.4)	24.2 (16.6)	9 (56.3)	10 (47.6)	5 (71.4)	14 (46.7)
15–18	18 (48.6)	55.4 (18.1)	42.9 (12.6)	27.1 (15.5)	7 (43.8)	11 (52.4)	2 (28.6)	16 (53.3)
**Ethnicity**								
Malay	21 (56.8)	57.8 (19.9)	41.2 (16.3)	29.2 (15.2)	6 (37.5)	15 (71.4)	4 (57.1)	17 (56.7)
Chinese	12 (32.4)	47.0 (9.9)	44.8 (11.6)	23.4 (17.4)	9 (56.3)	3 (14.3)	3 (42.9)	9 (30.0)
India	3 (8.1)	35.3 (16.8)	20.3 (12.1)	16.3 (7.6)	1 (6.3)	2 (9.5)		3 (10.0)
Other	1 (2.7)	50.0 (0)	16.0 (0)	4.0 (0)		1 (4.8)		1 (3.3)
**Normative need (NN)**								
No NN	8 (21.6)	45.0 (20.4)	35.8 (14.3)	24.9 (18.1)	3 (18.8)	5 (23.8)		8 (26.7)
NN	29 (78.4)	54.2 (16.8)	41.2 (16.4)	25.8 (15.6)	13 (81.3)	16 (76.2)	7 (100.0)	22 (73.3)

Pre-treatment, T0; Mid-treatment, T1; Post-treatment, T2; and standard deviation, SD.

**Table 2:** Changes in standardized Malaysian psychosocial impact of dental aesthetics questionnaire (PIDAQ) scores (*N* = 37).

	Standardized Scores				Differences in Standardized Scores			
	T0	T1	T2		T1−T0		T2−T0	
Scale Subscale	Mean (SD)	Mean (SD)	Mean (SD)	*p*-Value ^a^; Effect Size (η^2^)	Mean Diff (95% CI); *p*-Value ^b^	Effect Size (Descriptor)	Mean Diff (95% CI); *p*-Value ^b^	Effect Size (Descriptor)
**PIDAQ**	59.4 (20.2)	45.5 (18.1)	29.1 (18.1)	0.000 ^a^; 0.5	−13.9 (−22.0, −5.8); 0.000 ^b^	0.7 (medium)	−30.3 (−40.0, −20.5); 0.000 ^b^	1.5 (large)
DSC	22.2 (17.9)	39.4 (22.8)	61.5 (26.3)	0.000 ^a^; 0.5	17.2 (7.5, 27.0); 0.000 ^b^	1.0 (large)	22.1 (10.1, 34.0); 0.000 ^b^	2.2 (large)
SI	47.8 (27.4)	35.0 (22.5)	23.5 (19.0)	0.000 ^a^; 0.4	−12.8 (−22.0, −3.6); 0.004 ^b^	0.5 (medium)	−24.3 (−35.4, −13.2); 0.000 ^b^	0.9 (large)
PI	51.1 (23.2)	47.4 (22.2)	30.0 (19.3)	0.000 ^a^; 0.5	−11.7 (−20.1, −3.4); 0.004 ^b^	0.2 (small)	−29.2 (−39.7, −18.6); 0.000 ^b^	0.9 (large)
AC	51.0 (27.2)	36.1 (23.3)	20.9 (21.9)	0.000 ^a^; 0.4	−14.9 (−25.8, −3.9); 0.005 ^b^	0.2 (small)	−30.1 (−42.4, −17.7); 0.000 ^b^	1.1 (large)

^a^ *p* < 0.05, Repeated measures ANOVA; ^b^ *p* < 0.05, Post hoc pairwise comparisons; dental self-confidence, DSC; social impact, SI; psychological impact, PI; aesthetic concern, AC; Pre-treatment, T0; Mid-treatment, T1; Post-treatment, T2; and standard deviation, SD.

**Table 3:** Changes in Malaysian psychosocial impact of dental aesthetics questionnaire (PIDAQ) score scores by normative need.

	Within Group Differences				Between Group Differences		
		T1−T0	T2−T0	T2−T1	At T0	At T1	At T2
Group (n)	*p*-Value ^a^; Effect Size (η^2^)	Mean Diff (95% CI); *p*-Value ^b^; Effect Size (Descriptor)	Mean Diff (95% CI); *p*-Value ^b^; Effect Size (Descriptor)	Mean Diff (95% CI); *p*-Value ^b^; Effect Size (Descriptor)	Mean Diff (95% CI); *p*-Value ^c^; Effect Size (Descriptor)	Mean Diff (95% CI); *p*-Value ^c^; Effect Size (Descriptor)	Mean Diff (95% CI); *p*-Value ^c^; Effect Size (Descriptor)
No NN (8)	0.029 ^a^; 0.459	−9.3 (−24.6, 6.1); 0.307; 0.7 (medium)	−20.1 (−43.9, 3.6); 0.099; 1.0 (large)	−10.9 (−25.2, 3.4); 0.146; 0.9 (large)	−9.2 (−23.5, 5.0); 0.197; 0.5 (medium)	−5.4 (−18.4, 7.5); 0.401; 0.4 (small)	−1.0 (−14.0, 12.1); 0.883 0.1 (small)
NN (29)	0.000 ^a^; 0.526	−13.1 (−21.7, −4.4); 0.002 ^b^; 0.7 (medium)	−28.4 (−38.2, −18.6); 0.000 ^b^; 1.4 (large)	−15.3 (−24.5, −6.2); 0.001 ^b^; 0.8 (large)			

^a^ *p* < 0.05, Repeated measures ANOVA; ^b^ *p* < 0.05, Post hoc pairwise comparisons; ^c^ *p* < 0.05, paired *t*-tests; Pre-treatment, T0; Mid-treatment, T1; Post-treatment, T2; standard deviation, SD; normative need group, NN; and non-normative need group, No NN.

**Table 4:** Malaysian psychosocial impact of dental aesthetics questionnaire (PIDAQ) scores by proportions of perceived dental appearance from T0 to T2 (*N* = 37).

Perceived Dental Appearance	PIDAQ Score					
	T0		T1		T2	
	*n* (%)	Mean (SD)	*n* (%)	Mean (SD)	*n* (%)	Mean (SD)
Very poor	5 (13.5)	57.4 (20.5)				
Poor	11 (29.7)	59.3 (19.8)				
Average	19 (51.4)	45.4 (14.2)	6 (16.2)	44.8 (8.2)		
Good	1 (2.7)	50.0	24 (64.9)	38.1 (18.2)	20 (54.1)	29.6 (15.0)
Excellent	1 (2.7)	75.0	7 (18.9)	42.3 (12.3)	17 (45.9)	21.0 (16.1)
**Mean rank**		1.14		2.22		2.66
***p*** **-value ^a^**						0.000 ^a^

^a^ *p* < 0.05, Friedman test; Pre-treatment, T0; Mid-treatment, T1; Post-treatment, T2; and standard deviation, SD.

**Table 5:** Responsiveness of the Malaysian psychosocial impact of dental aesthetics questionnaire (PIDAQ) to changes based on the global health transition scale following orthodontic treatment (*N* = 37).

Scale Subscale	Responsiveness at T1					Responsiveness to Change at T2				
	*n*	T0	T1	Mean Change	Effect Size, d	*n*	T0	T2	Mean Change	Effect Size, d
		Mean (SD)	Mean (SD)	(SD) *p*-Value	(Descriptor)		Mean (SD)	Mean (SD)	(SD) *p*-Value	(Descriptor)
**A little improved ^a^**										
**PIDAQ**	16	42.9 (15.6)	37.7 (15.4)	−5.3 (13.4) 0.140	0.4 (Small)	7	51.4 (20.5)	25.1 (11.7)	−26.2 (25.4) 0.034 *	1.0 (Large)
DSC		7.2 (4.7)	9.4 (4.7)	2.3 (3.7) 0.027 *	0.6 (Medium)		7.0 (5.0)	14.6 (5.7)	7.6 (8.0) 0.046 *	1.0 (Large)
SI		11.8 (7.4)	10.4 (6.6)	−1.4 (5.7) 0.327	0.3 (Small)		15.9 (9.2)	7.3 (5.3)	−8.6 (9.9) 0.061	0.9 (Large)
PI		11.4 (4.7)	10.3 (4.8)	−1.2 (4.7) 0.332	0.2 (Small)		14.4 (6.9)	6.9 (4.1)	−7.6 (8.2) 0.050	0.9 (Large)
AC		2.9 (1.8)	2.5 (1.8)	−0.4 (1.8) 0.414	0.2 (Small)		4.1 (2.3)	1.6 (1.3)	−2.6 (3.0) 0.063	0.8 (Large)
**Much improved ^a^**										
**PIDAQ**	21	59.3 (16.2)	41.8 (16.5)	−17.6 (18.3) 0.000 *	1.0 (Large)	30	52.4 (17.4)	25.7 (16.9)	−26.7 (20.1) 0.000 *	1.3 (Large)
DSC		3.9 (3.5)	9.5 (6.1)	5.6 (6.5) 0.001 *	0.9 (Large)		4.9 (4.1)	14.8 (6.5)	9.9 (6.7) 0.000 *	1.4 (Large)
SI		18.0 (9.0)	11.8 (7.7)	−6.1 (7.6) 0.001 *	0.8 (Large)		15.2 (8.8)	7.6 (6.3)	−7.6 (8.5) 0.000 *	0.9 (Large)
PI		16.3 (5.3)	12.2 (5.6)	−4.0 (4.7) 0.001 *	0.9 (Large)		14.1 (5.3)	7.3 (4.8)	−6.9 (5.7) 0.000 *	1.2 (Large)
AC		5.0 (2.0)	3.2 (1.9)	−1.8 (2.2) 0.001 *	0.8 (Large)		4.1 (2.2)	1.7 (1.9)	−2.4 (2.3) 0.000 *	1.0 (Large)

^a^ Global health transition scale; * *p* < 0.05, paired *t*-test; dental self-confidence, DSC; social impact, SI; psychological impact, PI; aesthetic concern, AC; Pre-treatment, T0; Mid-treatment, T1; Post-treatment, T2; and standard deviation, SD.

**Table 6:** The minimal important differences (MID) of the Malaysian psychosocial impact of dental aesthetics questionnaire (PIDAQ) and dental self-confidence (DSC) subscale after orthodontic treatment.

Scale	Bland and Altman			MID	
	95% Limits of Agreement			Post-Treatment (T2)	
Subscale	Mean	Lower	Upper	Mean Change ^a^ (SD)	Standardized Effect Size ^b^ (Descriptor)
**PIDAQ**	−0.9	−10.3	8.5	−26.2 (25.4)	1.5 (large)
DSC	0.2	−5.3	5.6	7.6 (8.0)	2.2 (large)

^a^ anchor-based approach; ^b^ distribution-based approach; Post-treatment, T2; and standard deviation, SD.

## Data Availability

The data presented in this study are available on reasonable request from the corresponding author.

## Appendix A

**Table A1:** Psychosocial impact of dental aesthetic questionnaire (PIDAQ) scores of Malaysian sample.

	Orthodontic Patients [Current Study]			School Children [26]	Young Adults [24]
Scale	T0 (*N* = 37)	T1 (*N* = 37)	T2 (*N* = 37)	All (*N* = 901)	All (*N* = 524)
Subscale	Mean (SD)	Mean (SD)	Mean (SD)	Mean (SD)	Mean (SD)
**PIDAQ** ^b^	52.2 (17.8)	40.0 (15.9)	25.6 (15.9)	32.6 (16.2)	36.3 (17.1)
Dental self-confidence (DSC) ^a^	5.3 (4.3)	9.5 (5. 5)	14.8 (6.3)		
Reversed score DSC ^b^	18.9 (4.3)	14.5 (5. 5)	9.2 (6.3)	11.3 (5.4)	11.1 (5.2)
Social impact (SI) ^b^	15.3 (8.8)	11.2 (7.2)	7.5 (6.1)	9.0 (6.6)	11.1 (6.9)
Psychological impact (PI) ^b^	14.2 (5.6)	11.4 (5.3)	7.2 (4.6)	9.8 (5.4)	11.2 (5.3)
Aesthetic concern (AC) ^b^	4.1 (2.2)	2.89 (1.9)	1.7 (1.7)	2.4 (1.9)	2.9 (1.8)

^a^ Higher scores indicate good oral health related quality of life (OHRQoL), while lower scores indicate poor OHRQoL; ^b^ Higher scores indicate poor OHRQoL, while lower scores indicate good OHRQoL; pre-treatment, T1; mid-treatment, T2; and post-treatment, T2.
